# ﻿Larval and female descriptions of *Mejicanotrichia* Harris & Holzenthal, 1997 (Trichoptera, Hydroptilidae, Leucotrichiinae) from Mexico

**DOI:** 10.3897/zookeys.1111.77413

**Published:** 2022-07-11

**Authors:** Mauricio Ramírez-Carmona, Rafael Barba-Álvarez, Atilano Contreras-Ramos, Gerardo Rivas

**Affiliations:** 1 Posgrado en Ciencias del Mar y Limnología, Universidad Nacional Autónoma de México (UNAM), Cd. Universitaria, 04510 Mexico City, Mexico Universidad Nacional Autónoma de México (UNAM) Mexico City Mexico; 2 Departamento de Zoología, Instituto de Biología, UNAM, Universidad Nacional Autónoma de México, Cd. Universitaria, 04510 Mexico City, Mexico Universidad Nacional Autónoma de México (UNAM) Mexico City Mexico; 3 Departamento de Biología Comparada, Facultad de Ciencias, Universidad Nacional Autónoma de México (UNAM), Cd. Universitaria, 04510 Mexico City, Mexico Universidad Nacional Autónoma de México (UNAM) Mexico City Mexico

**Keywords:** Biodiversity, caddisflies, immature stages, madicolous habitat, Neotropics, water quality

## Abstract

*Mejicanotrichia* Harris & Holzenthal, 1997 is a small genus of Hydroptilidae (Trichoptera), which consists of seven species, six of them distributed in Mexico, and one more in Guatemala. Larval descriptions of only two species (*M.blantoni* and *M.estaquillosa*) were previously known, as well as only females of three species (*M.blantoni*, *M.estaquillosa*, and *M.tamaza*) previously described. The present study provides descriptions of the larvae of *M.harrisi and M.tridentata*, as well as a description of the female of *M.harrisi*. Identification keys for adult males, known females, and known larvae are also provided. This work aims to incorporate more information into the taxonomy of the genus, its ecology, and facilitate additional characters of potential use in future phylogenetic studies.

## ﻿Introduction

Hydroptilidae represents the most diverse family of the order Trichoptera, currently with 2,570 species recorded worldwide (Morse et al. 2019) and 946 species distributed in the Neotropics (Holzenthal and Calor 2017). The genus *Mejicanotrichia* Harris & Holzenthal, 1997 was established to segregate species related to *Mejicanotrichiablantoni* (Flint, 1970), previously included in the genus *Alisotrichia* Flint, 1964. The genus contains seven recognized species, six of them distributed in Mexico, and one in Guatemala (Holzenthal and Calor 2017). The first larval description and illustration of *Mejicanotrichia* were provided by Wiggins (1996) within the genus *Alisotrichia*. Bowles et al. (1999) specified that the larva actually belonged to the genus *Mejicanotrichia*, in particular to *M.estaquillosa* Harris & Holzenthal, 1997; they also described the larva of *M.blantoni* and discussed the phylogenetic position of the genus within the subfamily Leucotrichiinae. Later, Santos et al. (2016) performed a phylogenetic analysis with morphological data that confirmed that *Mejicanotrichia* belongs to the monophyletic subfamily Leucotrichiinae and the tribe Alisotrichiini.

The larvae of this genus are characterized by having numerous, broad, and largely colorless setae on the dorsoventrally flattened body, with oval shaped sclerites on the prosternum and the divided meso- and metathorax. The body exhibits an ornamentation with pigmented points on the surface of the thorax and abdomen; the legs are of the same size and shape, with tarsal claws well developed, as well as anal prolegs prominent, square shaped, each with a simple large claw (Wiggins 1996; Bowles et al. 1999). A larval case is absent until the final instar; it resembles a seed and is attached to large, submerged rocks in madicolous habitats. The larvae inhabit running waters with high flow and are sometimes associated with waterfall systems (Harris and Holzenthal 1997; Bueno-Soria 2010). Adults are small (2–4 mm body length), the head bears three ocelli, antennae are simple; wings are narrowed and attenuated with a reduced venation; males present first wings modified with patches of scales except in *M.harrisi* Bueno-Soria & Barba-Álvarez, 1999 and *M.rara* Bueno-Soria & Barba-Álvarez, 1999 (Harris and Holzenthal 1997). The tibial spur formula is 0-2-4 (Flint 1970; Harris and Holzenthal 1997) and male sternite IX features a deep notch (Harris and Holzenthal 1997; Bueno-Soria and Barba-Álvarez 1999). Adult individuals are generally active during the day, found on vegetation and substrate along waterfalls or turbulent currents (Bueno-Soria 2010). Larvae of only two species, *M.blantoni* and *M.estaquillosa*, are known; similarly, females of only three species, *M.blantoni*, *M.estaquillosa*, and *M.tamaza* (Flint, 1970) have been described.

Thus, the aim of this study is to contribute to the knowledge of *Mejicanotrichia*, particularly larvae and females of the Mexican species, as well as to provide an environmental characterization of the larval habitat of the genus. We gladly dedicate this contribution to Dr. Ralph W. Holzenthal of the University of Minnesota, with our admiration as one of the main experts of Neotropical aquatic entomology, particularly in recognition to his dedication for the study of caddisfly biodiversity in Latin America.

## ﻿Materials and methods

Specimens of *Mejicanotrichiaharrisi* and *M.tridentata* (Bueno-Soria and Hamilton 1986) were collected in their type localities and its surroundings. Larvae and metamorphotypes (Milne 1938) were collected manually with thin entomological tweezers on large boulders. Adults were collected with an entomological aspirator during the day on boulders at the streams; likewise, the collections were made with an UV light trap. The specimens were preserved in absolute ethanol. Larvae were mounted with glycerin on temporary slides with concave depressions for observation. Drawings were performed under a Zeiss optical microscope using a clear-field camera with magnifications of 10×, 16×, and 40×. The pencil drawings were scanned and edited on Adobe Illustrator CC. In order to clear the genitalia and allow species level identification, adult entire specimens were placed in 10% potassium hydroxide (KOH) at room temperature for seven hours, then rinsed in a solution of acetic acid and distilled water to neutralize KOH. After clearing, specimens were stored in absolute ethanol. For observation of adults, the entire specimen was placed on a concave slide in pure glycerin. Several larval specimens were fixed in absolute ethanol and then coated with gold (Bozzola and Russell 1999) for scanning electron microscopy (JEOL JSM 6360-LV).

The morphological terminology used is based on Marshall (1979) for adult females and Wiggins (1996) for larvae. Taxonomic identification was based on Flint (1970), Bueno-Soria and Hamilton (1986), Harris and Holzenthal (1997), Bueno-Soria and Barba-Álvarez (1999), and Bueno-Soria (2010). The environmental parameters of the streams were evaluated in situ. Water temperature and dissolved oxygen (OD) were measured with an YSI model 54ARC oximeter; pH was evaluated with Tetra brand reactive strips; and total hardness was estimated by means of a Hach the titration kit (test 5B) given as calcium and magnesium carbonates (1 gpg = 17.1 mg CaCO_3_/l).

## ﻿Taxonomic descriptions

### ﻿Larva

#### ﻿ Mejicanotrichia harrisi

Taxon classificationAnimaliaTrichopteraHydroptilidae

Bueno-Soria & Barba-Álvarez, 1999

7B9A07D2-17E0-5979-BA69-A42AAECEDCD9

Figs 1A–C , 2A–D

##### Material examined.

25 larvae (IN-TR-00221). Mexico, Guerrero, Tonalapa del Río, Tonalapa River, near the Atlmolonga “balneario” (780 m a.s.l., 18°20'57.05"N, 99°42'10.12"W), 25 January 2020; leg. M. Ramírez-Carmona and O. Lagunas-Calvo.

##### Diagnosis.

**Figure 1.**
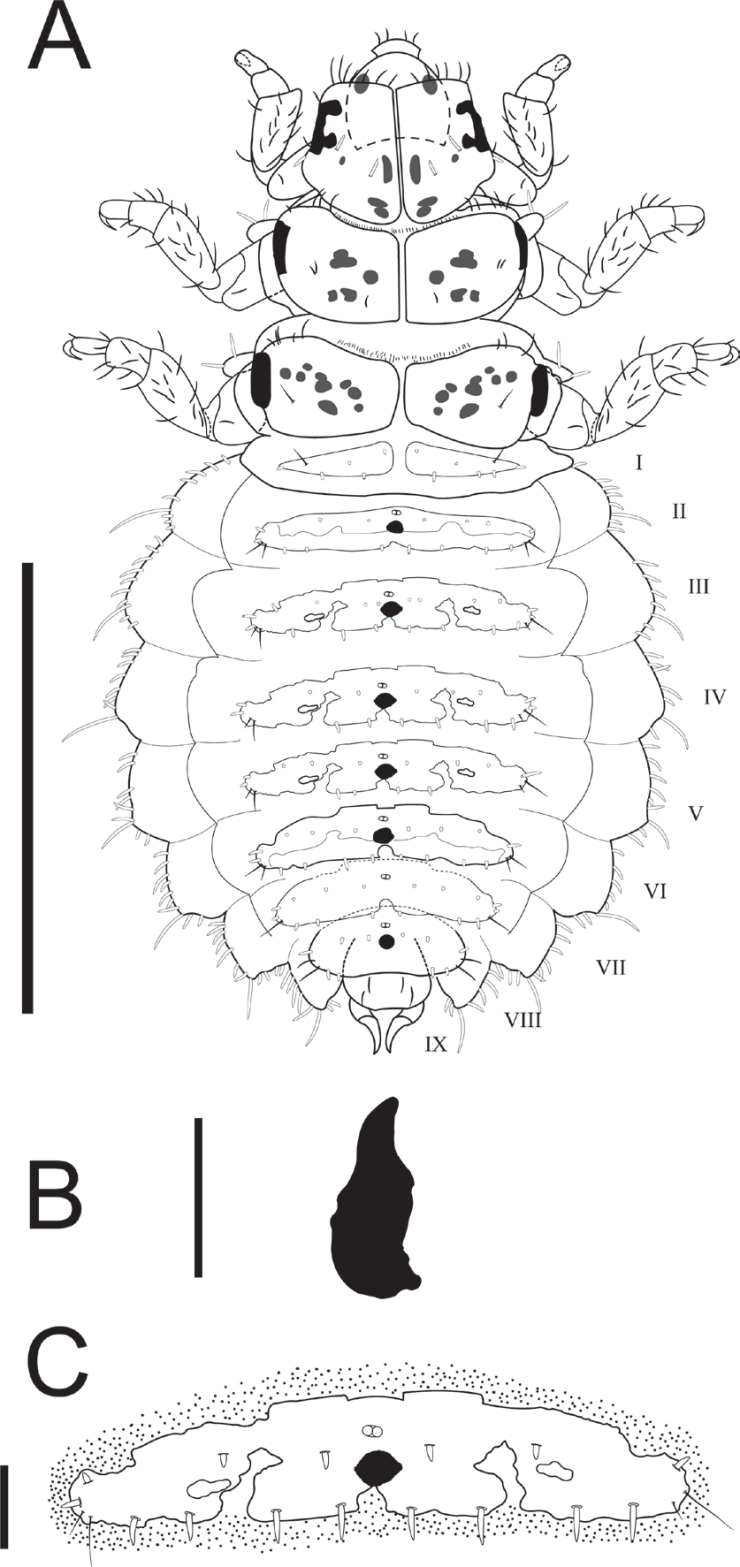
Larva of *Mejicanotrichiaharrisi* Bueno-Soria & Barba-Álvarez, 1999 **A** habitus, dorsal **B** propleural sclerite **C** tergite of abdominal segment V, dorsal. Scale bars: 1 mm (**A**); 80 µm (**B**); 130 µm (**C**).

Abdomen mainly membranous with presence of abdominal tergites and fine pigment spots (Fig. 1A). Propleural sclerite with irregular shape (Fig. 1B) compared to *M.tridentata*, also the abdomen is wider in appearance. The size of the mature larvae of *M.harrisi* (2.1 mm) is smaller compared to the other species of *Mejicanotrichia*. The propleural sclerite has the form of a “serrated tooth” unlike the other species of the genus (Figs 1B, 4B; Bowles et al. 1999). Additionally, the abdominal dorsal tergites are wider and shorter than in *M.tridentata*, which exhibits a larger number of setae over the tergites than *M.harrisi*.

##### Description.

**Figure 2.**
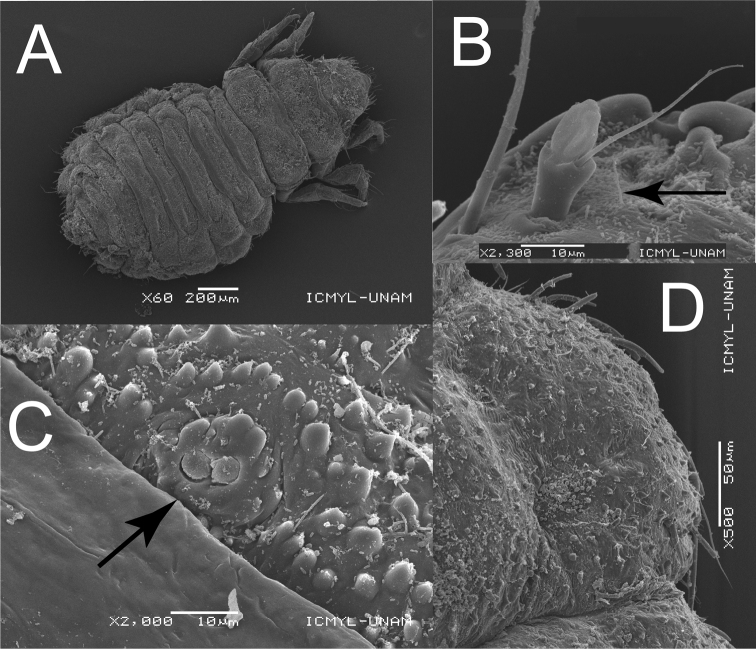
Scanning electron micrographs of larva of *Mejicanotrichiaharrisi* Bueno-Soria & Barba-Álvarez, 1999 **A** habitus, dorsal **B** antenna **C** lacunae of abdominal segment V **D** lateral projection of abdominal segment V. Scale bars: 200 µm (**A**); 10 µm (**B, C**); 50 µm (**D**).

Dorsoventrally depressed body, range length: 1.9–2.1 mm, covered extensively by colorless and thick setae (Fig. 1A). Dorsum covered almost entirely by fine pigments spots (ornamental) (Fig. 1C), which give it the appearance of “sandpaper” (Fig. 2A). Larval case absent until before pupation.

***Head.*** Dark brown, prognathous, without visible ecdysial sutures. Antennae simple (Fig. 2B).

***Thorax.*** Pro-, meso-, and metanotum divided by a median ecdysial line (Fig. 1A), with a lateral process with two thick and colorless setae except on pronotum. Pronotum anteriorly elongated, subsequently widened, longer than meso- and metanotum, covering much of the head; anterolateral corners folded towards the ventral region. The anterior pronotal margin with a row of thick and opaque setae. Propleural sclerite well developed and strongly dark, subtriangular (Fig. 1B). Prothorax with a pair of ventral sclerites, each with an oval to subrectangular shape. Meso- and metathorax covered with thick, short, colorless setae. Lateral margins thickly darkened, appearing with a longitudinal bar (Fig. 1A). Anterior margins of both nota with a row of opaque setae, in a smaller proportion than the pronotum. First pair of legs slightly shorter than the others. The three pairs of legs each with two rows of fine and moderately long setae on tibiae and tarsi, with well-developed tarsal claws.

***Abdomen.*** Long and wide, gradually tapering posteriorly. Venter with thick and extremely short setae irregularly distributed on surface. Segments I–VIII with well developed, short and wide tergites, covering much of each notum, those on segment I differing noticeably in size and shape from remainder. All tergites with thick and short colorless setae. Tergite I divided in two by a median line, with two thin and dark setae on the anterior margin. Tergites II–VII with medial lacunae and two fine and dark setae in the posterolateral margin (Figs 1A, C, 2C). Posterior and anterior margins of tergites with thick, short, and colorless setae, well distributed along both margins. Fine pigment spots more evident on integument around tergites (Fig. 1C). Middle of anterior margin of tergites III–VI, with two flattened projections. Lateral projections of abdomen without tergites, but lateral margins bear a continuous row of slightly thick, long, colorless setae (Fig. 2D); one seta noticeably longer than the others, arising at apex of each lateral projection. Segment IX strongly narrowed, posterior margin with four thick and opaque setae, two rows of thick setae in the middle. Anal prolegs prominent, cylindrical, projecting posteriorly, with well-developed anal claws curving ventrally (Fig. 1A).

##### Comments.

The specimens were collected at a water temperature of 19 °C; with pH between 7.8–8.4; water presented a hardness of 171 mg CaCO3/l; the dissolved oxygen was 7.6 mg/l and 89% of oxygen saturation.

### ﻿Female

#### ﻿ Mejicanotrichia harrisi

Taxon classificationAnimaliaTrichopteraHydroptilidae

Bueno-Soria & Barba-Álvarez, 1999

21336A5B-614B-5A4C-B799-FBCB94A43B02

Fig. 3A–D

##### Material examined.

5 females (IN-TR-00220). Mexico, Guerrero, Tonalapa del Río, Tonalapa River, near the Atlmolonga “balneario” (780 m a.s.l, 18°20'57.05"N, 99°42'10.12"W), 25 January 2020; leg. M. Ramírez-Carmona and O. Lagunas-Calvo.

##### Diagnosis.

Unmodified antennae, hindwings lacking patches of scales, as in *M.rara*, with a body length of 2 mm. Abdominal segment VI without sternite process.

##### Description.

**Figure 3.**
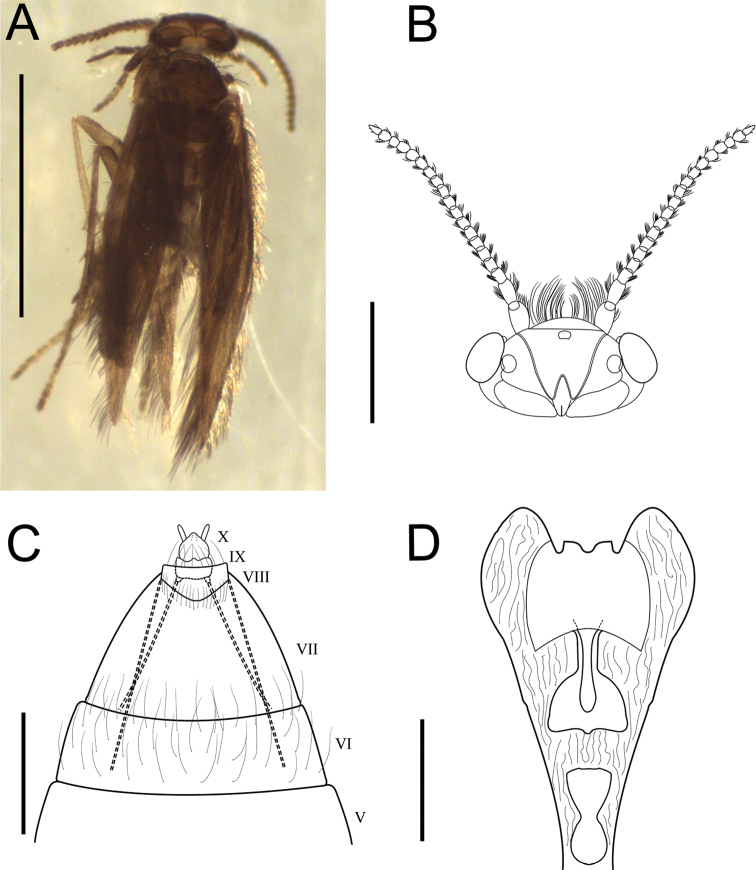
Female specimen of *Mejicanotrichiaharrisi* Bueno-Soria & Barba-Álvarez, 1999 **A** habitus, dorsal **B** head, dorsal **C** segments VI-X of abdomen, ventral **D** bursa copulatrix ventral. Scale bars: 1 mm (**A**); 800 µm (**B**); 200 µm (**C**); 30 µm (**D**).

Dark brown coloration (Fig. 3A). Body. Range length: 2.3–2.5 mm.

***Head.*** Antennae simple, 17-segmented, scape slightly longer than flagellum; three ocelli present (Fig. 3B).

***Thorax.*** Wings with reduced venation, mesoscutellum with a transverse line and subrectangular metascutellum. Tibial formula (0, 2, 4). Legs unmodified.

***Abdomen.*** Segment VII elongated, without processes on sternite. Segment VIII short and ring-shaped, with a fringe of setae on posterior margin and a pair of apodemes extending anteriorly. Segment IX short, with pair of apodemes originating on posterolateral margin and extending anteriorly just before anterior margin of segment VII. Segment X rounded apically, with pair of lateral papillae (Fig. 3C). Bursa copulatrix mostly membranous, with pair of short and truncate lobes extended posteriorly; medially with keyhole-shaped opening and a shield-shaped sclerite (Fig. 3D).

##### Comments.

The specimens exhibit a keyhole-shaped opening in the bursa copulatrix, which occurs in the other species of the genus. On the other hand, females of *M.harrisi* differ from those of other species because of the presence of two short and membranous lobes that extend posteriorly, as well as for having a shield-shaped posterior sclerite (Fig. 3D).

### ﻿Larva

#### ﻿ Mejicanotrichia tridentata

Taxon classificationAnimaliaTrichopteraHydroptilidae

(Bueno-Soria & Hamilton, 1986)

877C3D90-4240-5CBF-B6B5-5443A7779304

Figures 4A–C , 5A–D

##### Material examined.

15 larvae (IN-TR-00222). Mexico, Chiapas, Ixhuatán, 95 km 2.8 N Ixhuatán, tributary of the Teapa River (409 m a.s.l., 17°18'41.06"N, 93°0'17.78"W), 18 April 2019; leg. M. Ramírez-Carmona, O. Lagunas-Calvo and G. Rivas-Lechuga.

##### Diagnosis.

**Figure 4.**
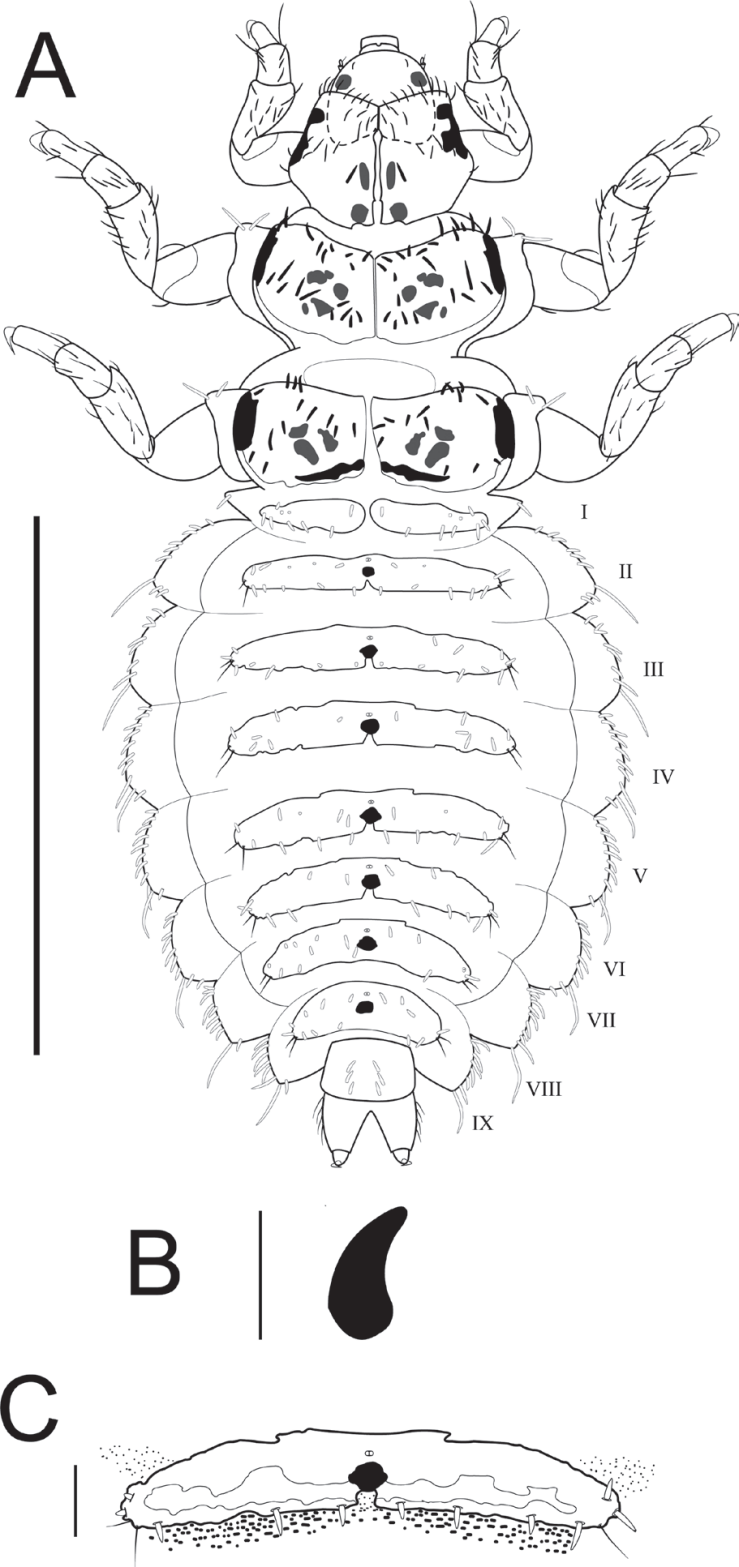
Larva of *Mejicanotrichiatridentata* (Bueno-Soria & Hamilton, 1986) **A** habitus, dorsal **B** propleural sclerite **C** tergite of abdominal segment V, dorsal. Scale bars: 1 mm (**A**); 65 µm (**B**); 80 µm (**C**).

Body mostly membranous ventrally. Thorax reddish-brown with dark spots dorsally. Abdominal tergites with dark and irregular spots (Fig. 4A). Conspicuous, dark, subtriangular sclerites on pleural area (Fig. 4B). A subtriangular and curved propleural sclerite is notoriously distinct from that of other species of the genus (Figs 1B, 4B; Bowles et al. 1999: figs 4–7). Abdominal dorsal tergites are long and narrow, with a larger number of setae than in *M.harrisi*. In addition, these tergites have a pigmentation pattern that is evenly distributed.

##### Description.

Body dorsoventrally depressed, range length 2.0–2.3 mm, widely covered with thick, long, and colorless setae (Fig. 4A). Body mostly covered dorsally with fine pigments spots, appearing as “sandpaper” (Fig. 4C). Larval case absent until before pupation.

***Head.*** Ocherous-brown, prognathous, without visible ecdysial sutures.

**Figure 5.**
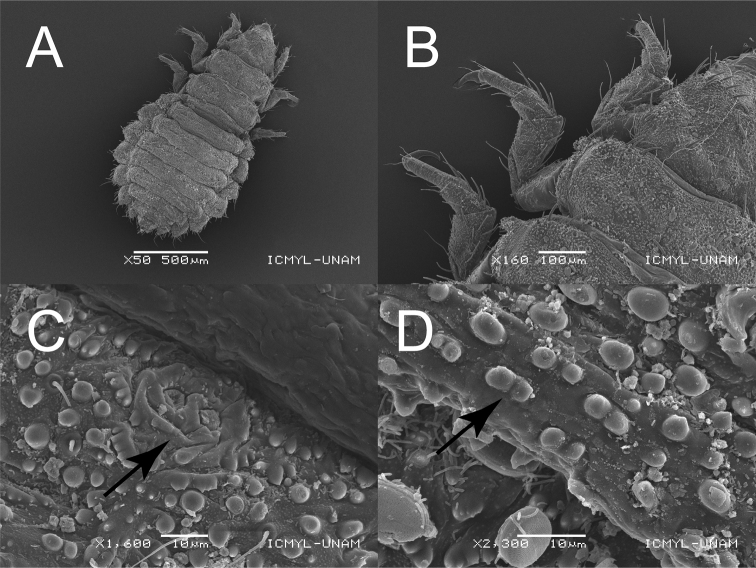
Scanning electron micrographs of larva of *Mejicanotrichiatridentata* (Bueno-Soria & Hamilton, 1986) **A** habitus, dorsal **B** left thoracic legs, dorsal **C** lacunae of abdominal segment V **D** dark ornamental pigments of abdominal tergites. Scale bars: 500 µm (**A**); B: 100 µm (**B**); C: 10 µm (**C, D**).

***Thorax.*** Pro-, meso-, and metanotum divided longitudinally by a medial ecdysial line. Three thoracic nota each with two lateral processes, which have two thick and opaque setae. Pronotum widening posteriorly. Anterior margin of pronotum with a ridge of thick setae, anterolateral corners folding ventrally. Anterior portion of pronotum slightly covering back of the head (Figs 4A, 5A). First pair of legs slightly smaller, tibiae and tarsi of all legs each with two rows of fine setae on dorsal region (Fig. 5B). Two oval sclerites behind the insertion of the legs on each sternite. Anterior margin of meso- and metanotum with row of thick setae, separated at least twice the basal diameter of setae. Lateral margins short and darkened, appearing as longitudinal bars. Mesonotum covered extensively with thick and short setae.

***Abdomen.*** Long and widened, narrowing posteriorly. Ventral region with irregularly distributed, thick, short setae. Segments I–VIII with dorsal tergites, largely covering dorsum of each segment; first sclerite divided longitudinally. Tergites II–VII with lacunae in middle and beyond posterior margin (Fig. 5C); with two fine and dark setae on posterolateral margins, and well-developed thick setae on anterior and posterior margins (Fig. 4C). Anterior margins of tergites II–VI each with a slight notch in the middle; fine pigment spots linearly grouped beyond posterior margin of tergites (Fig. 5D). Abdominal projections without tergites; lateral margins with row of thick and prominent setae, one of which noticeably longer than rest. Segment IX short and shield-shaped, with setae on posterior margin, as well as six setae medially on segment. Anal prolegs projected caudally, cylindrical, with well-developed claws.

##### Comments.

The specimens were collected at a water temperature of 25 °C; with pH between 7.8–8.4; water presented a hardness of 136.8 mg CaCO3/l; the dissolved oxygen was 4.5 mg/l and 57% of oxygen saturation.

## ﻿Identification keys

### ﻿Key to adult males of *Mejicanotrichia* (after Bueno-Soria and Barba-Álvarez 1999)

**Table:** 

1	Phallus with apical or subapical spines	**2**
–	Phallus without apical or subapical spines (Bueno-Soria and Barba-Álvarez 1999: fig. 8)	***M.harrisi***
2	Phallus with three pairs of spines apically (Harris and Holzenthal 1997: fig. 10D, E)	**3**
–	Phallus with two pairs or less of apical or subapical spines (Harris and Holzenthal 1997: fig. 8D, E)	**4**
3	Phallus apically with three pairs of elongate lateral spines and a central spine (Harris and Holzenthal 1997: fig. 10D, E)	***M.estaquillosa***
–	Phallus apically with three pairs of short spines and a pair of short lateral spines subapically (Harris and Holzenthal 1997: fig. 3D, E)	***M.blantoni***
4	Phallus apically with two pairs of elongate spines	**5**
–	Phallus apically without spines (Harris and Holzenthal 1997: fig. 6D, E; Bueno-Soria and Barba-Álvarez 1999: fig. 8)	**6**
5	Phallus apically with a pair of elongate, weak spines laterally, pair of thin spines mesally and subapically with a pair of spicule bearing tergites (Harris and Holzenthal 1997: fig. 5D, E)	***M.tamaza***
–	Phallus apically with two pairs of elongate spines, without a subapical pair of spicules bearing tergites (Harris and Holzenthal 1997: fig. 8D, E)	***M.tridentata***
6	Phallus subapically with a pair of lateral spines, with ejaculatory duct emerging between spines (Harris and Holzenthal 1997: fig. 6E)	***M.trifida***
–	Phallus subapically with a pair of thin, elongate spines laterally, without ejaculatory duct emerging between spines (Bueno-Soria and Barba-Álvarez 1999: fig. 4)	***M.rara***

### ﻿Key to known females of *Mejicanotrichia*

**Table:** 

1	Bursa copulatrix with two membranous lobes (Fig. 3D)	**2**
–	Bursa copulatrix with one membranous lobe (Harris and Holzenthal 1997: fig. 4D)	***M.tamaza***
2	Bursa copulatrix with keyhole-structure, with keyhole-shaped sclerite mesally (Fig. 3D)	**3**
–	Bursa copulatrix without keyhole-structure, with oval sclerite mesally (Harris and Holzenthal 1997: fig. 4F)	***M.estaquillosa***
3	Bursa copulatrix with shield-shaped sclerite (Fig. 3D)	***M.harrisi***
–	Bursa copulatrix without shield-shaped sclerite (Harris and Holzenthal 1997: fig. 4A)	***M.blantoni***

### ﻿Key to known larvae of *Mejicanotrichia*

**Table:** 

1	Fine pigments in posterior margin of abdominal tergites without pattern in pairs (Fig. 2C)	**2**
–	Fine pigments in posterior margin of abdominal tergites with pattern in pairs (Figs 4C, 5D)	***M.tridentata***
2	Propleural sclerite with rounded shape, claw-shaped or triangle-shaped (Bowles et al. 1999: figs 6, 7)	**3**
–	Propleural sclerite not round-shaped, instead serrate tooth shaped (Fig. 1B)	***M.harrisi***
3	Tergites without a blotched pattern over the body, propleural sclerite triangle-shaped (Bowles et al. 1999: fig. 6)	***M.blantoni***
–	Tergites with a blotched pattern over the body, propleural sclerite claw-shaped (Bowles et al. 1999: fig. 7)	***M.estaquillosa***

## ﻿Discussion

**Figure 6.**
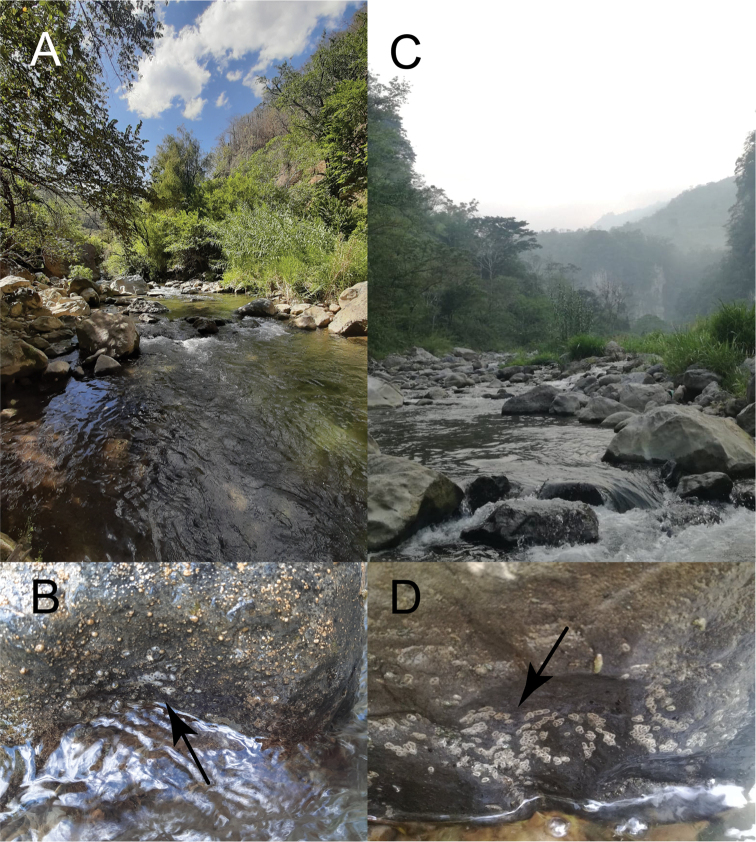
Collecting sites and cases of *Mejicanotrichia* in madicolous microenvironments **A** Tonalapa river, Guerrero **B***M.harrisi*, pupal cases attached to rock, indicated by arrow **C** tributary of the Teapa river, Chiapas **D***M.tridentata*, pupal cases attached to rock, indicated by arrow.

The genus *Mejicanotrichia* is a Mesoamerican taxon with species distributed in a restricted fashion, both regarding a specific microhabitat, as well as a narrow geographical area. Knowledge of these species is practically limited to morphology of the adult males. The present work increases the knowledge of the larvae and we also contribute the description of the female of one species: *Mejicanotrichiaharrisi* was originally described from the Temazcalapa River, in the state of Guerrero, with specimens collected between 1994 and 1995. We attempted to recover specimens from the original locality, but the stream was found to be completely dry. It was at a tributary of the Tonalapa River, 10 km away from the type locality, that adult (females and males) and larval specimens of *M.harrisi* were collected (Fig. 6A, B). In the case of *M.tridentata*, it was originally described within the genus *Alisotrichia* and collected in 1983 in Chiapas state; in the present study, we were able to recover immature stages at the type locality (Fig. 6C, D), thus allowing us to complete the taxonomic information of the species.

The larvae of *M.harrisi*, *M.tridentata*, and those described in previous studies differ from each other in the shape of the propleural sclerite (Figs 1B, 4B; Bowles et al. 1999), and by having different abdominal dorsal tergites, with *M.tridentata* having these longer and narrower than *M.harrisi*, as well as exhibiting a larger number of setae distributed on the tergites (Fig. 4A). Similarly, the fine pigment spots associated with the dorsal abdominal tergites present a group arrangement in the form of “horizontal bars” (Figs 4C, 5D). Although Bowles et al. (1999) do not mention abdominal dorsal tergites as distinctive characters between species (*M.blantoni* and *M.estaquillosa*), in the present work the dorsal sclerites on the abdomen were considered as distinctive characters between *Mejicanotrichia* species.

Species delimitation in Trichoptera is based entirely on primary characters presented by male genitalia, as these are conspicuous and complex (Holzenthal et al. 2007), whereas the female genitalia are much simpler, thus offering a smaller number of characters (Nielsen 1980; Holzenthal et al. 2007). However, the females of *M.harrisi* treated in the present study differ from those of the other species because they exhibit two short membranous lobes, which extend into the posterior region, as well as a posterior sclerite in form of a “shield” (Fig. 1D).

Some of the ecological affinities of the genus have resulted in morphological adaptations to the habitat, as referred to by Marshall (1979) and Bowles et al. (1999); some of the modifications resulted in a dorsoventrally flattened body, well-developed abdominal tergites and sturdy climbing legs. Larvae of *Mejicanotrichia* were found on the surface of large rocks, in high flowing environments and associated with river waterfalls (Bowles et al. 1999; Bueno-Soria 2010); and also in a madicolous habitat, referred to by Bowles et al. (1999) as the developmental environment for some hydroptilid larvae (Fig. 6B, D). In this habitat, coexistence with other genera associated with *Mejicanotrichia*, such as *Scelobotrichia*, *Leucotrichia*, and *Alisotrichia* (Santos et al. 2016), occurs.

Much of the flora of madicolous habitats is restricted to microalgae, which are mostly diatoms or patches of filamentous algae (Vaillant 1956; Sinclair and Marshall 1986), so the larvae of *Mejicanotrichia* may belong to the “scraper” feeding group. On the other hand, the larvae of the genus do not present a case in the early stages, and it is not until the pupation stage that one is made. This case consists entirely of silk and resembles a seed attached to the surface of the rock (Wiggins 1996; Bowles et al. 1999; Bueno-Soria 2010). Otherwise, there are no studies (ecophysiological and ecological) that demonstrate the role played by hardness (mg CaCO_3_/l) on trichopteran biology. Nevertheless, the data obtained in the field represent a clear example of the overlap of distribution of *Mejicanotrichiaharrisi and M.tridentata* with karst substrate in the country (Espinasa-Pereña 2007). In the two localities of study (Ixhuatán and Atlmolonga), the hardness estimates correspond to hard water (USGS scale), and in two of them hardness data were obtained at two different times, which reflected similar values, being slightly higher in the dry season. Therefore, this presupposes a possible relationship between water hardness and the presence of *Mejicanotrichia*.

## Supplementary Material

XML Treatment for Mejicanotrichia harrisi

XML Treatment for Mejicanotrichia harrisi

XML Treatment for Mejicanotrichia tridentata
